# Serum markers of apoptosis decrease with age and cancer stage

**DOI:** 10.18632/aging.100069

**Published:** 2009-07-14

**Authors:** Nilay Kavathia, Alka Jain, Jeremy Walston, Brock A. Beamer, Neal S. Fedarko

**Affiliations:** ^1^ Division of Geriatric Medicine and Gerontology, Department of Medicine, Johns Hopkins University, Baltimore, MD 21224, USA; ^2^ Current address: Thomas Jefferson University, Philadelphia, PA 19107, USA; ^3^ Current address: University of Maryland, School of Medicine, Baltimore, MD 21201, USA

**Keywords:** apoptosis, serum markers, immunosenescence, aging, cancer, cytochrome c

## Abstract

The physical manifestations of aging reflect a loss of homeostasis that effects molecular, cellular and organ system functional capacity. As a sentinel homeostatic pathway, changes in apoptosis can have pathophysiological consequences in both aging and disease. To assess baseline global apoptosis balance, sera from 204 clinically normal subjects had levels of sFas (inhibitor of apoptosis), sFasL (stimulator of apoptosis), and total cytochrome c (released from cells during apoptosis) measured. Serum levels of sFas were significantly higher while sFasL and cytochrome c levels were lower in men compared to women. With increasing age there was a decrease in apoptotic markers (cytochrome c) and pro-apoptotic factors (sFasL) and an increase in anti-apoptotic factors (sFas) in circulation. The observed gender differences are consistent with the known differences between genders in mortality and morbidity. In a separate cohort, subjects with either breast  (n = 66) or prostate cancer (n = 38) exhibited significantly elevated sFas with reduced sFasL and total cytochrome c regardless of age. These markers correlated with disease severity consistent with tumor subversion of apoptosis. The shift toward less global apoptosis with increasing age in normal subjects is consistent with increased incidence of diseases whose pathophysiology involves apoptosis dysregulation.

## Introduction

Apoptosis is an evolutionary conserved program that leads to cell death. Apoptotic cell death plays a role in normal development (e.g. - embryogenesis, morphogenesis) and in maintaining adult homeostasis (e.g. - immune response resolution, tissue remodeling, elimination of damaged/dysfunctional cells) [1,2]. The physical manifestations of aging reflect a loss of homeostasis that effects molecular, cellular and organ system functional capacity. As a sentinel homeostatic pathway, changes in apoptosis can have patho-physiological consequences in aging. For example, too much apoptosis can yield tissue degeneration [3-6], while too little apoptosis allows either dysfunctional cells to  accumulate  or differentiated  immune cells  to persist [7-9]. Thus, cellular maintenance protocols involve a delicate balance in pro- and anti-apoptotic factors/signals.

Fas is a cell-surface receptor that transduces apoptotic signals from another cell-surface receptor Fas ligand, FasL [10,11]. Fas and FasL have also been observed as soluble molecules. Soluble Fas arises from alternatively spliced mRNA [9,10] and all variants of sFas inhibit apoptosis induced by FasL [12,13]. FasL can undergo proteolytic cleavage to liberate a 26 kDa soluble form of the molecule [14]. The physiological role of sFasL in the regulation of apoptosis remains unclear as both stimulatory [15,16] and inhibitory [17,18] activity has been reported. Cytochrome c has a well defined role in triggering apoptosis and as a marker of apoptosis [19], though it was recently shown that cytochrome c exists in a complex in serum with leucine-rich alpha-2-glycoprotein-1 which altered immunoreactivity [20]. In order to assess the global balance of systemic markers of apoptosis, we developed an immunoassay to measure total serum levels of cytochrome c and determined the distribution and levels of sFas, sFasL and total cytochrome c in serum from a large clinically defined normal group. In addition, we used the same surrogate markers of apoptosis to characterize their levels in a group well characterized as having altered apoptosis (i.e. - cancer subjects).

## Results

We determined serum levels of sFas in 204 normal subjects. For all subjects, values for fasting glucose, thyroid panel, and calculated BMI were within the normal range.

**Figure 1.**
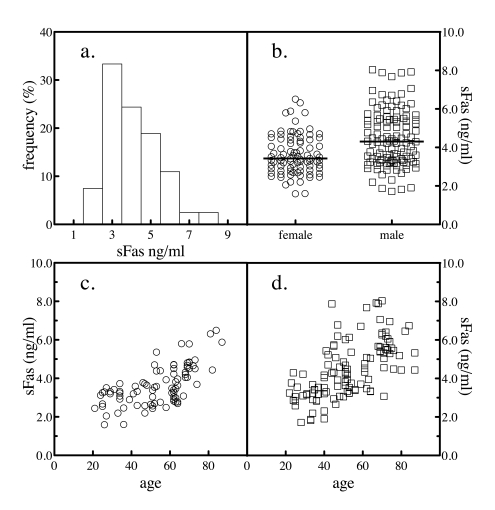
Serum sFas levels. The levels of sFas in 204 normal subjects was determined by sandwich ELSA. The frequency distribution of the values across the subjects was analyzed (**a**). The levels of sFas by gender were plotted (**b**). The sample population was segregated by gender and the levels of serum sFasL as a function of donor age for female (**c**) and male (**d**) subjects were plotted.

**Table 1.** Serum levels of apoptosis biomarkers. Biomarker levels were compared by gender. The association of serum levels with donor age was analyzed by Spearman correlation. ^a^ Mann Whitney U-test comparing serum values in females versus males. ^b^ Correlation coefficient (r) for Spearman nonparametric correlation analysis of serum biomarker levels and donor age. ^c^ P value for Spearman nonparametric correlation analysis of serum biomarker levels and donor age.

	sFas (pg/ml)		sFasL (pg/ml)		Cytochrome c (μg/ml)	
	female	male	female	male	female	male
mean±SD median (range)	3625±1019 3424 (1592-6498)	4475±1459 4303 (1710-8026)	94.6±22.3 97.9 (45.8-139.4)	91.2±20.8 92.4 (40.6-145.6)	0.712±0.206 0.663 (0.24-1.33)	0.703±0.420 0.566 (0.13-2.22)
gender ^a^	p < 0.0001		p = 0.13		p = 0.053	
age r ^b^	0.651	0.647	-0.534	-0.337	-0.719	-0.855
p value ^c^	< 0.0001	< 0.0001	< 0.0001	< 0.001	< 0.0001	< 0.0001

The mean value for sFas was 4107 ± 1352 pg/ml. When the frequency distribution of serum values was analyzed by histogram, a slight hook at the high end was evident (Figure 1a). The results were stratified by gender to further study the distribution. For the samples obtained from the 94 female donors, the mean donor age was 53 and ranged from 21 to 87, while for the 110 male donors, the mean age was 52 and ranged from 22 to 88. Serum levels of sFas were significantly higher in males than in females, comparing by a Mann Whitney test (Figure 1b and Table 1). Mean BMI values were 22.6 ± 1.4 and 22.1 ± 1.6 kg/m2 for women and men, respectively. The difference by gender in sFas levels was still significant after controlling for BMI. When sFas levels were plotted versus the age of the subject, the reason for the high-end hook to the distribution of normal values became apparent. Both genders exhibited an age-dependent increase in sFas values with age (Figure 1c and d).

**Figure 2.**
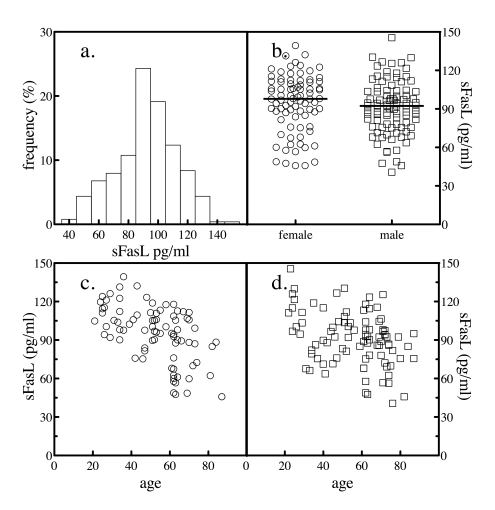
Serum sFasL levels. The levels of sFasL in 204 normal subjects was determined by sandwich ELSA. The frequency distribution of the values across the subjects was analyzed (**a**). The levels of sFasL in all subjects as a function of gender were plotted (**b**). The sample population was segregated by gender and the levels of serum sFasL as a function of donor age for female (**c**) and male (**d**) subjects were plotted.

The serum levels of sFasL were determined in the same subjects. The mean value for sFasL was 92.8 ± 21.5 pg/ml. When the distribution of serum values was analyzed by histogram, a slight hook at the low end was evident (Figure 2a). Again, the results were stratified by gender to further study the distribution. Serum levels of sFasL were not significantly different between genders (Figure 2b and Table 1). Plotting sFasL levels versus the age of the subject revealed that both genders exhibited an age-dependent decrease in sFasL values (Figure 2c and d).

**Figure 3.**
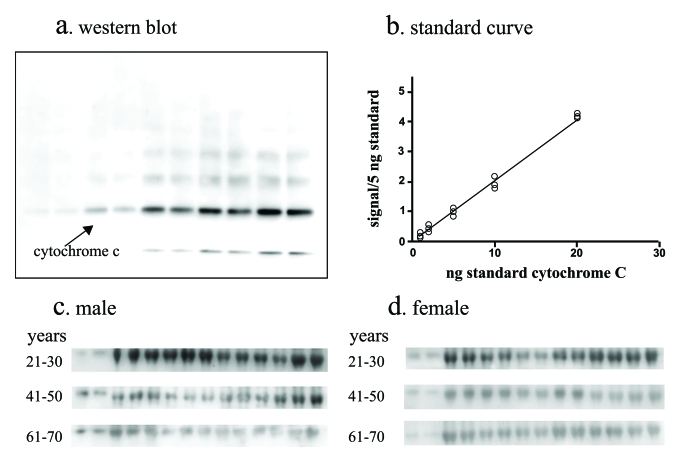
Total cytochrome c assay. A quantitative western blot assay was developed to measure total cytochrome c in serum. The assay employed denaturing and reducing conditions to disrupt cytochrome c binding to carrier proteins in serum. The assay utilized serial dilutions of purified cytochrome-c resolved by SDS PAGE and western blotting (**a**) to generate standard curves (**b**) by digitally imaging and quantifying the chemiluminescent signal and serum from men (**c**) and women (**d**) were analyzed in parallel. Standards and serum samples were analyzed in duplicate.

While a role for sFas as an anti-apoptotic factor is accepted in the literature, the pro-apoptotic role of sFasL is more equivocal [15-18]. A third marker for apoptosis was developed. Cytochrome c release from the mitochondria is a sentinel signal initiating apoptosis [21] and serum levels of cyt-c have been used as a marker of apoptosis [22,23]. However, cytochrome c is bound to in serum to leucine-rich alpha-2-glycoprotein-1 which can mask antibody epitopes, potentially interfering with immunoassay quantification [20]. We developed a quantitative western blot using purified cytochrome c to generate a standard curve and interpolate unknown concentrations from serum samples that had been denatured and reduced thereby disrupting binding complexes and enabling the quantification of total cytochrome c levels (Figure 3).

**Figure 4.**
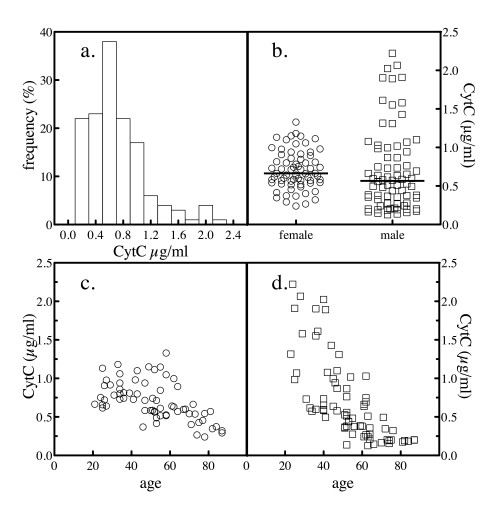
Serum total cytochrome c levels. The levels of total cytochrome c in 204 normal subjects were determined as depicted in Figure 3. The frequency distribution of the values across the subjects was analyzed (**a**). The levels of total cytochrome c in all subjects by gender was plotted (**b**). The sample population was segregated by gender and the levels of serum cytochrome c as a function of donor age for female (**c**) and male (**d**) subjects were plotted.

 The mean value for serum levels of total cytochrome c was 0.71 ± 0.42 μg/ml. The frequency distribution of serum values was analyzed by histogram and a nonparametric distribution was evident (Figure 4a). When the results were stratified by gender, the difference in mean (and median) values by gender were not significant (Figure 4b and Table 1). Plotting total cytochrome c levels versus the age of the subject revealed that both genders exhibited an age-dependent decrease in total cytochrome c, though the slopes appeared to be different (Figure 4c and d).

**Figure 5.**
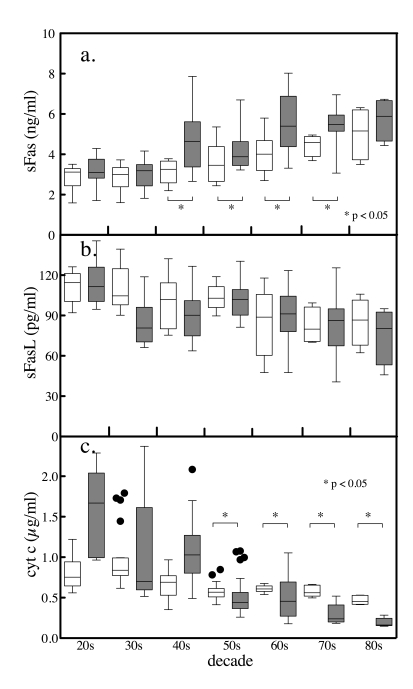
Age and gender differences in serum sFas,. sFasL and total cytochrome c levels. The serum levels of the apoptotic biomarkers were segregated by gender and by decade. Tukey box and whiskers plots (female clear boxes, male shaded boxes) of sFas (**a**), sFasL (**b**) and total cytochrome c (**c**) depicting the top, bottom, and line through the middle of the box correspond to the 75th percentile (top quartile), 25th percentile (bottom quartile), and 50th percentile (median) respectively. The error bar-like whiskers depict 1.5 x the interquartile range and the solid circles represent outliers. Comparisons between genders were performed conservatively by Mann Whitney U-test.

Because of the nonparametric distribution of these apoptotic markers, the association of serum levels with donor age was analyzed conservatively by Spearman nonparametric correlation (Table 1). Significant correlations of subject age versus serum marker levels were observed. sFas in serum correlated positively with increasing age among females, among males and among the two combined. In contrast, FasL and total cytochrome c correlated negatively with age. Segregating serum samples by gender and by decade of life enabled statistical comparison of gender values by decade using a nonparametric Mann Whitney test. Between the ages of 41 and 80, females had significantly lower levels of the anti-apoptotic marker sFas compared with men (Figure 5a). The serum levels of the potentially pro-apoptotic sFasL, although higher on average in females, were not significantly different then those in men over the seven decades (Figure 5b). The apoptosis marker cytochrome c exhibited levels that were different between men and women from perimenopausal ages onward (Figure 5c).

The observed shifts in the balance of pro- and anti-apoptotic factors (sFasL and sFas, respectively) and the apoptosis marker (cytochrome c) with age are consistent with decreased net apoptosis with increasing age. Neoplasm growth and tumor progression rely in part on blocking apoptosis [24-26]. Serum from a group of women with breast cancer (n = 66) and men with prostate cancer (n=38) were analyzed for sFas, sFasL and total cytochrome c and the distribution of the values compared with age and gender-matched normal values (Table 2). sFas levels were significantly elevated in both breast and prostate cancer. In contrast, sFasL and cytochrome c levels were significantly reduced in both breast and prostate cancer.

**Figure 6.**
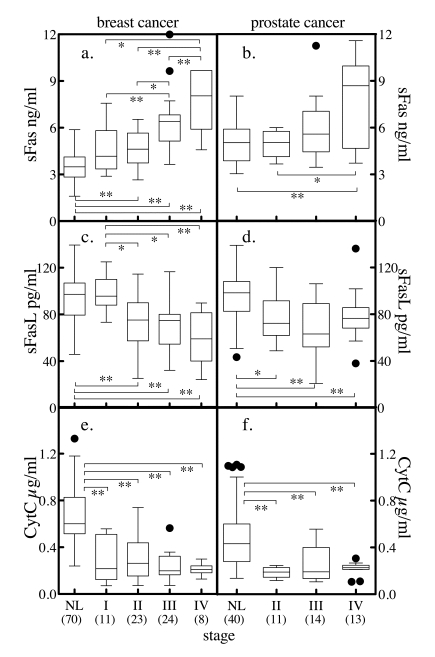
Serum markers of apoptosis and tumor stage. Subjects with breast cancer (**a, c, e**), or prostate cancer (**b, d, f**) were stratified by stage and the distribution of sFas (**a, b**), sFasL (**c, d**) and cytochrome c (**e, f**) stratified by staging was determined. The solid horzontal bars depict the median values.  For breast cancer, stage I tumor size (T) < 2 cm across and cancer cells have not spread to axillary lymph nodes (N). For stage II, T < 2 cm across and the cancer has spread to the lymph nodes under the arm (N positive) or T is 2 to 5 cm and N is negative. In stage III, T > 5 cm or it has spread to other lymph nodes or tissues near the breast. Stage IV is metastatic cancer. The convention for prostate cancer staging was that in stage I, cancer is found in the prostate only. In stage II, cancer is more advanced than in stage I, but has not spread outside the prostate. In stage III, cancer has spread beyond the outer layer of the prostate to nearby tissues. Stage IV is characterized by distant metastasis. Comparison between group median values was performed by Mann Whitney t-test, where * = p < 0.05, ** = p < 0.005, *** = p < 0.0001. Numbers in parenthesis indicate number of subjects in each group.

The association of cancer stage groupingswith apoptosis markers was investigated for breast and prostate cancer. The breast cancer serum values were segregated by stage where stage I is small localized tumors with no spreading to axillary lymph nodes; stage II disease has larger tumors and potential spread to the lymph nodes; stage III disease has spread to other lymph nodes or tissues near the breast; while stage IV is metastatic cancer. For prostate cancer, stage II cancer is localized within the prostate but palpable, stage III cancer has broken through the covering of the prostate but is still regional, and stage IV cancer has spread to other tissues. When the distribution of sFas, sFasL and cytochrome c were profiled by stage using Tukey box plots, discrete patterns were observed (Figure 6).

Serum sFas levels increased with increasing stages of breast cancer (Figure 6a). While stage I disease was not significantly different from normal, stages II, III, and IV were significantly elevated relative to the normal. The more advanced stage III disease was significantly elevated compared to normal and earlier stages, and significantly lower compared to stage IV disease. Metastatic disease (stage IV) was significantly elevated compared with all other stages and had a median value ~2-fold higher then normal and stage I breast cancer. Serum sFas levels in prostate cancer exhibited a similar trend of increasing median values with increasing stage. However, only stage IV disease was significantly different from both normal and stage I disease (Figure 6b).

Serum sFasL levels in breast cancer decreased with increasing stage, with more advanced stages (II, III and IV) significantly different from normal and stage I (Figure 6c). With prostate cancer, sFasL levels decreased significantly between normal and stages II, II and IV (Figure 6d). Similarly, serum cytochrome c levels were significantly reduced between normal and stages I through IV of breast cancer (Figure 6e) and between normal and stages II, II and IV of prostate cancer (Figure 6f). Thus, subjects with cancer have higher anti-apoptotic factors (sFas) in circulation and less proapoptotic factors (sFasL, cytochrome c) in circulation.  Also, the more advanced the cancer, the larger the change in circulating levels.

**Table 2.** Serum levels of apoptosis biomarkers in cancer. ^a^ Age in years ± standard deviation. A subset of the normal female and male groups were age- and gender-matched to the specific cancers. ^b^ Mann Whitney U-test comparing serum values in breast and prostate cancer subjects to age- and gender matched normal subjects.

	female NL	BCA	male NL	PCA
n	70	66	40	38
Age (years)^a^	-	62 ± 14	-	66 ± 9
sFas (pg/ml)				
mean±SD	3585±918	5202±1732	5023±1309	6249±2324
median	3490	4831	5038	5587
range	1603-5877	2651-11990	3048-8026	3462-11580
U-test^b^	p < 0.001		p < 0.05	
sFasL (pg/ml)				
mean±SD	94.4±20.1	75.3±26.2	89.0±19.6	69.7±22.0
median	97.3	75.2	92.2	62.2
range	45.9-139.4	15.6-125.0	40.6-130.3	19.4-127.7
U-test^b^	p < 0.0001		p < 0.0001	
Cytochrome c (μg/ml)				
mean±SD	0.673±0.266	0.27±0.14	0.458±0.243	0.23±0.09
median	0.601	0.24	0.406	0.21
range	0.239-1.329	0.07-0.74	0.128-1.039	0.09-0.046
U-test^b^	p < 0.0001		p < 0.0001	

## Discussion

Apoptosis, originally believed to be a process with only negative effects, now is recognized to balance the beneficial potential of eliminating damaged cells against the pathological effects of deleterious cell death (e.g. neurodegenerative disease) [27]. Failures in apoptosis can contribute to the senescent cell phenotype as well as rogue cell proliferation [28]. It has been shown that apoptosis is an important cellular defense mechanism in maintaining genetic stability, and centenarians who have aged successfully possess cells that are more prone to apoptosis [29]. The major age related disease leading to mortality is cardiovascular disease.  Studies have shown that apoptotic cell death effect cardiac tissue, and in addition, cells that avoid apoptosis participate in the progression of atherosclerosis [30,31]. Cancer, another leading cause of mortality, arises from neoplastic progression through avoidance of apoptosis [32].  In addition, dysregulation of Fas/FasL mediated apoptosis can contribute to the pathogenesis of pulmonary [33,34] liver [35], and neoplastic [36] fibrosis.

Studies with mice having Fas/FasL mutations suggest that that a major function of Fas-mediated apoptosis is the elimination of activated immune cells from the peripheral circulation [37]. Similarly, humans with autoimmune lymphoproliferative syndrome have mutations in Fas [38,39]. Maintenance of Fas apoptosis signaling is a crucial feature for successful immune aging [40]. In young immune fit individuals, stimulation of T cells leads to upregulation of Fas, FasL, and Fas/FasL engagement-induced apoptosis signaling causing cell death which eliminates the majority of T cells that are activated in response to a stimulus, thereby preventing the accumulation of autoreactive T cells. An age-related impairment of Fas/FasL mediated apoptosis is believed to contribute to compromised regulation of the immune system and immunosenscence [28]. The age related shift in favor of reduced apoptosis (higher sFas with lower sFasL and total cytochrome c) may contribute to reduced clearance of immune cells leading to a state of chronic inflammation [27]. A chronic inflammatory state may underlie a number of pathologies including cancer [41], cardiovascular disease [42,43], diabetes mellitus [44], frailty [45,46], osteoporosis [47], rheumatoid arthritis [48], and cognitive disorders such as Alzheimers and Parkinson's disease [49-51]. It is of note that the pro-inflammatory marker interleukin-6 appears to be protective against apoptosis [52-55], its serum levels are known to increase with increasing age [56] and have an inverse correlation with Fas-induced apoptosis [57].

In the immune system, Fas and FasL are involved in down-regulation of immune reactions as well as in T cell-mediated cytotoxicity [58]. In cancer, malignant cells inhibit the expression of membrane-bound Fas and express FasL which triggers tumor-infiltrating lympho-cyte apoptotic cell death [59]. In contrast to their membrane-bound forms, soluble sFas and sFasL exhibit different patterns. The levels of sFas and sFasL have been measured independently in separate studies in different populations of normal subjects [60,61] and subjects with breast cancer [62-64] and prostate cancer [65,66].  Similarly, serum cytochrome c has been measured as a marker of apoptotic cell death [19,67] and in cancer [21,68-70]. In general, serum Fas was elevated in cancer patients while sFasL levels were elevated or reduced, depending on the cancer group. Interpretation of published results on serum cytochrome c are complicated by the recent observation that cytochrome c exists in a complex with leucine-rich alpha-2-glycoprotein-1 in serum which alters immunoreactivity [20]. Thus, it is not clear whether studies measuring cytochrome c directly in serum are quantifying a free (unbound) pool or a pool reflecting some combination of free and complexed cytochrome c. In the current study, levels of 500 ng/ml total cytochrome c were measured on average in the normal population, which is at least 10-fold higher then published values [20,71,70].

In a study of 204 clinically defined normal subjects, serum levels of sFas increased while sFasL and total cytochrome c decreased with increasing subject age.  In addition, the age-related elevation of sFas was significantly higher, while total cytochrome c was significantly lower in males from their 40's and 50's onward. This is the first report describing the distribution of these multiple markers in a single, well-defined normal population. The healthy normal group had extensive exclusion criteria to minimize confounding due to age-related conditions. Aging is a loss of homeostasis and pathologies traditionally referred to as age-related diseases (e.g. - cardiovascular disease, cancer, diabetes, Alzheimer's, osteoporosis) can be considered as manifestations of fast aging [72]. Given the correlations observed between donor age and the apoptosis markers in the normal healthy group, the expansion of the study group to include age-related diseases (whose serum values would reflect fast aging) might be expected to broaden the differences in these serum markers.

The observed shift in the balance to decreased apoptosis may contribute to age-associated increases in diseases characterized by failure of normal apoptosis (e.g. - cancer, arthritis, cardiovascular disease). Indeed, in both breast and prostate cancer, correlative data on serum sFas, sFasL and total cytochrome c that were consistent with a shift toward decreasing apoptosis were also observed in the current study. Finally, many observations indicate that women have a longer life expectancy than men, that mortality and morbidity are higher in men than in women and this gender difference is constant in cardiovascular disease, cancer and dementia [73]. The observed gender differences in apoptosis markers - higher sFas and reduced sFasL and total cytochrome c - which are of indicative of dysregulated apoptosis would be consistent with the increased mortality and morbidity in men.

## Methods

Subjects. Approval for the study protocol was acquired from the local institutional review board and informed consent was obtained from all patients. Sera from clinically defined normal patients were obtained from a commercial serum bank (SeraCare Life Sciences Inc., Oceanside, CA) as well as from the Johns Hopkins Bayview Medical Center General Clinical Research Center (JHBMC).  The JHBMC normal group was obtained from an existing serum bank using samples from which all patient identifiers were removed. For this study, inclusion criteria as a normal serum donor included measures within the normal range for fasting glucose (< 100 mg/dl), TSH (0.5 - 2.1 mIU/mL), BMI (20 - 25 kg/m^2^) as well as a physical assessment by a physician. Exclusionary criteria included a previous history of hypertension, heart disease, diabetes mellitus, renal or hepatic dysfunction, cancer, or any chronic inflammatory condition (e.g., rheumatoid arthritis). Sera from a group of 104 cancer subjects consisting of 66 females with breast cancer and 38 males with prostate cancer were obtained from a serum repository. Blood was drawn at time of diagnosis, prior to initiation of treatment.

Serum biochemical measures. Blood samples were drawn in the morning after an overnight fast. Serum biochemical measurements included sFas and sFasL by sandwich enzyme immunoassay technique (R&D, Systems, Minneapolis, MN). The assay performance characteristics in the laboratory for sFas were a sensitivity of 22.4 pg/ml, an intra-assay coefficient of variance of 2.48% and an inter-assay coefficient of variance of 6.06% and for sFasL were a sensitivity of 7.2 pg/ml, an intra-assay coefficient of variance of 3.64% and an inter-assay coefficient of variance of 6.87%.

Total cytochrome c assay. Cytochrome c protein stan-dard (equine heart) was obtained from EMD Chemicals (Gibbstown, NJ). A mouse monoclonal anti-cytochrome c unconjugated antibody was obtained from Invitrogen (Carlsbad, CA). Goat anti-mouse IgG conjugated to horseradish peroxidase was obtained from Kirkgaard & Perry (Gaithersburg, MD). NuPAGE 4-12 % Bis -Tris 1.5 mm X 15 well polyacrylamide gels, NuPAGE antioxidant and See blue pre-stained standards were obtained from Invitrogen. Super Signal West Dura Extended Duration Substrate was obtained from Thermo Fisher Scientific Inc. (Waltham, MA).

Serum samples, after being reduced with 10 mM DTT and diluted in gel sample buffer (1:10), were resolved by Nu PAGE 4-12% Bis Tris gel.  8μl of diluted and reduced sample was loaded onto the gel for each sample. Purified equine heart cytochrome c was used to generate a standard curve at 20, 10, 5, 2, and 1 ng/well. After electrophoresis, samples were transferred to nitrocellulose membrane following standard conditions. After a 1-h incubation in blocking solution (TBS-Tween+5% non fat powdered milk) at room temperature on rotary shaker, a mouse monoclonal anti-cytochrome c antibody was added at a dilution of 1: 2000 and incubated over night at 4 c on a rotary shaker. The nitrocellulose membrane was washed in TBS-Tween three times for 5 minutes each and then goat anti-mouse IgG conjugated to horseradish peroxidase diluted to 1:10,000 in TBS-Tween was added and incubated for 2 hrs at room temperature. Following removal of second antibody solution, the membrane was washed three times with TBS -Tween and exposed to the chemiluminiscent enzyme substrate for 5 minutes. Signals were captured, digitized and analyzed using a Kodak GEL Logic 2200 Imaging System (Carestream Health Inc., Rochester, NY).

Statistical analysis. Comparisons between groups were performed conservatively using the Mann Whitney nonparametric test. The association of sFas, sFasL or cytochrome c with donor age was analyzed using the conservative Spearman nonparametric correlation test. All statistical calculations were carried out using GraphPad Prism version 5.00 for MacOS (GraphPad Software, San Diego CA).
